# Molecular dissection of pathway components unravel atisine biosynthesis in a non-toxic *Aconitum* species, *A. heterophyllum* Wall

**DOI:** 10.1007/s13205-016-0417-7

**Published:** 2016-04-18

**Authors:** Varun Kumar, Nikhil Malhotra, Tarun Pal, Rajinder Singh Chauhan

**Affiliations:** Department of Biotechnology and Bioinformatics, Jaypee University of Information Technology, Waknaghat, Solan, HP 173234 India

**Keywords:** Atisine, *A. heterophyllum*, Steviol, qPCR, Biosynthesis, Correlations, Transcriptome

## Abstract

*Aconitum heterophyllum* is an important component for various herbal drug formulations due to the occurrence of non-toxic aconites including marker compound, atisine. Despite huge pharmacological potential, the reprogramming of aconites production is limited due to lack of understanding on their biosynthesis. To address this problem, we have proposed here the complete atisine biosynthetic pathway for the first time connecting glycolysis, MVA/MEP, serine biosynthesis and diterpene biosynthetic pathways. The transcript profiling revealed phosphorylated pathway as a major contributor towards serine production in addition to repertoire of genes in glycolysis (G6PI, PFK, ALD and ENO), serine biosynthesis (PGDH and PSAT) and diterpene biosynthesis (KO and KH) sharing a similar pattern of expression (2-4-folds) in roots compared to shoots vis-à-vis atisine content (0–0.37 %). Quantification of steviol and comparative analysis of shortlisted genes between roots of high (0.37 %) vs low (0.14 %) atisine content accessions further confirmed the route of atisine biosynthesis. The results showed 6-fold increase in steviol content and 3–62-fold up-regulation of all the selected genes in roots of high content accession ascertaining their association towards atisine production. Moreover, significant positive correlations were observed between selected genes suggesting their co-expression and crucial role in atisine biosynthesis. This study, thus, offers unprecedented opportunities to explore the selected candidate genes for enhanced production of atisine in cultivated plant cells.

**Electronic supplementary material:**

The online version of this article (doi:10.1007/s13205-016-0417-7) contains supplementary material, which is available to authorized users.

## Introduction

Medicinal plants are the prolific repositories of specialized metabolites having great commercial value as drugs used in the treatment of various disorders. Traditionally, they have been used as a major source of medication for the treatment of various ailments. Later on, with the advent of chromatographic separations and advancement in organic chemistry, efforts began to isolate and identify the bioactive compounds in plants and strive to synthesize the compounds in vitro. Owing to the sophisticated structures of most natural products, it became a formidable task to synthesize them, and thus, the natural products remain gleaned from the native medicinal plants (Barnes and Prasain 2005). Further, the rising demand of natural products facilitated over-harvesting of several medicinal plants and as a result the reckless collection reduced their populations in natural habitats, thus falling into the category of endangered plant species.

*Aconitum heterophyllum* Wall, commonly known as Atis, is one of the rare medicinal herb in the Himalayan region of India found between 2400 and 3600 m altitude which has been listed as “critically endangered medicinal herb” by IUCN (International Union for Conservation of Nature and Natural Resources) (IUCN 1993; Nautiyal et al. 2002). It has been banned for export on 30th March, 1994 by Govt. of India, Ministry of Commerce through their circular Public Notice No. 47 (PN)/92–97, and was further revised through Notification no. 24 (RE-98)/1997–2002 (Shah 2005).

Generally, *Aconitum* has been considered as a mysterious herb due to its both healing and death causing properties. Among the reported ~300 species of *Aconitum*, *A. heterophyllum* is the only non-toxic species with therapeutic potential (Chauhan 2006). The non-toxic roots of *A. heterophyllum* are used for the treatment of chronic fever, throat infection, indigestion, flatulence, diarrhea, arthritis, abdominal distension, dyspepsia, stomachache and coughs (Lather et al. 2010; Mitra et al. 2001; Negi et al. 2011; Prasad et al. 2012; Sojitra et al. 2013; Subramoniam et al. 2013). It has also been used in many herbal formulations, viz. Balachaturbhadrika churna, Rodhrasava, Siva Guika, Mahavisagarbha taila, Lakasminarayana rasa and Pancatikta guggulu ghrta (Nariya et al. 2011; Shyaula 2011). Moreover, it has also been used as an aphrodisiac and tonic (Semwal et al. 2009). The pharmacological properties of *A. heterophyllum* are attributed to the non-toxic active constituents, i.e., aconites which including atisine comprise the major alkaloid constituents of the plant (Malhotra et al. 2014). Therefore, gazing into the upsurge of interest in aconites and threat to its extinction, efforts need to be directed towards increased production of aconites in *A. heterophyllum*. In recent years, various strategies have been employed in other plants to modulate the level of desired metabolites but use of genetic intervention strategy has been suggested as the solution to such problem (Kumar et al. 2015a, b, e; Sharma et al. 2015). Therefore, rational engineering of aconites should be executed to redirect the carbon flux towards aconites production in *A. heterophyllum*. However, that requires a thorough knowledge of the entire biosynthetic pathway of aconites in *A. heterophyllum*.

**Fig. 1:**
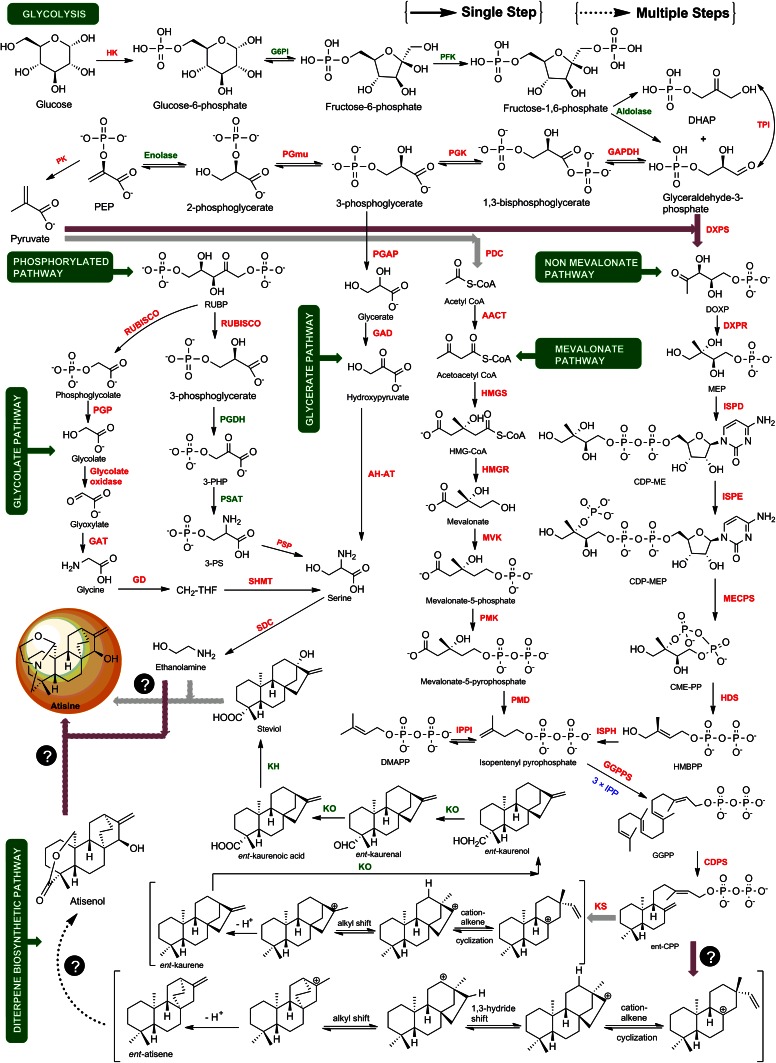
Plausible biosynthetic pathway leading to atisine in *A. heterophyllum*. The metabolic network has been constructed by including glycolysis, phosphorylated, glycerate, glycolate, MVA/MEP and diterpene biosynthetic pathways. Question marks indicate missing enzymes with no available information. *Green color* represents the positions of enzymes identified as candidate genes in atisine biosynthesis. Abbreviations are elaborated in supplementary Table 4

Atisine is a diterpenoid alkaloid. The biosynthesis of diterpenoid alkaloids (DAs) has been scarcely investigated and so far there is dearth of reports available on their biosynthesis in *A. heterophyllum*. However, the atisine-type DAs corresponding to the basic skeleton of atisane-type diterpenes have been isolated from the *Spiraea japonica* complex (Hao et al. 2003). Atisine is thought to be produced from mevalonate (MVA) and non-mevalonate (MEP) pathways leading to the formation of diterpene precursor geranylgeranyl diphosphate (GGPP) (Malhotra et al. 2014), but information is not yet available on the biosynthesis of atisine beyond GGPP. To fill this gap, we constructed a metabolic network for the first time which showed the coordination and connecting links between different pathways integrating into atisine production to provide a more robust view of atisine biosynthesis in *A. heterophyllum* (Fig. 1).

Endorsement of plausible metabolic pathway for atisine in *A. heterophyllum* must be the important next step. For this, use of radioactive/stable isotope tracers required time and resource demanding experiments (Stephanopoulos 2012). Thus, in search of facile methods, we used a new tactic of ‘omics’ which provided the genomics inspired opportunities to correlate the patterns of gene expression with specific metabolites that not only pursued the genetic control of metabolite production, but also validated the biosynthetic route of target metabolites (Askenazi et al. 2003).

To enable this, quantitative real time-PCR (qRT-PCR) technique has emerged as a powerful tool that serves to target specific pathways over a wide range of experimental conditions and estimated the modulations in gene expression patters with amazed sensitivity and accuracy (Asters et al. 2014; Kumar et al. 2015c).

Taken together, this work probes the atisine biosynthesis in *A. heterophyllum* for the first time and also explores the participating pathways with metabolic bottlenecks associated with atisine production by understanding the correlation of all concerned genes vis-à-vis target metabolite content.

## Materials and methods

### Plant material

Two years old *A. heterophyllum* plants designated as high and low content accessions (Malhotra et al. 2014) were collected from the Himalayan Forest Research Institute at Shilaru, Himachal Pradesh, India (2450 m altitude, 31°23′ N, 77°44′ E) by permission of Dr. Sandeep Sharma, HFRI, Shimla (H.P.). The plants were maintained at Jaypee University of Information Technology (Waknaghat, Himachal Pradesh; 31°59′ N, 77°13′ E; 1700 m altitude). The samples were segregated into root and shoot tissues, frozen in liquid nitrogen and immediately stored at −80 °C for further analyses.

### Construction of atisine biosynthetic pathway

A plausible pathway for atisine biosynthesis in *A. heterophyllum* was constructed through a bio-retrosynthetic approach which involves the arrangement of biosynthetic pathway steps from target metabolite to its precursor (Bachmann 2010). In this approach, by considering the structure of atisine and known chemical transformations, we identified the precursors of atisine, i.e., GGPP and serine which then served as the starting points for another retrobiosynthetic precursor. The process was reiterated until a suitable starting pathway, i.e., glycolysis was found which began the synthesis of target molecule, i.e., atisine.

### Shortlisting of paralogs

The shortlisting of paralogs was carried out to avoid ambiguity among multiple paralogs of the selected genes found in the transcriptomes of *A. heterophyllum*. Nucleotide sequences of selected genes for glycolysis, serine biosynthesis and diterpene alkaloid biosynthesis were retrieved from the transcriptomes of *A. heterophyllum* available at the URL: http://14.139.240.55/NGS/download.php (Pal et al. 2015). The functionally characterized sequences of each selected gene were obtained from the public databases and subjected to blastn/tblastn in NCBI blast (http://blast.ncbi.nlm.nih.gov/Blast.cgi) as query sequences against the transcriptomic sequences of corresponding genes as subject sequences. The paralogs showing maximum homology with the functionally characterized sequences were selected and further screened on the basis of transcript abundance in differential metabolite phenotypes.

### Genomic DNA and total RNA isolation

The shoots of *A. heterophyllum* plants were used for genomic DNA isolation as per the method reported by Murray and Thompson (1980). For total RNA isolation, the root and shoot samples of *A. heterophyllum* plants designated as high and low content accessions were collected and homogenized in prechilled pestle and mortar using liquid nitrogen. To 100 mg of each powdered sample, 1 mL TRIzol reagent (Sigma, USA) was added and incubated at room temperature for 5 min. To the above samples, 0.2 mL chloroform was added and vortexed followed by incubation at room temperature for 2–3 min. The samples were then centrifuged at 12,000×*g* for 15 min at 4 °C. The upper aqueous layer was then transferred to a fresh microfuge tube and 0.5 mL isopropanol was added to precipitate the RNA. The samples were then incubated at 4 °C for 10 min and centrifuged at 12,000×*g* for 10 min at 4 °C. The resulting pellet was washed twice with chilled 70 % ethanol and finally dissolved in 60 μL of RNase-free water. The quality of isolated RNA was checked on 1 % (w/v) agarose gel stained with ethidium bromide and visualized by using gel documentation system (Alpha Innotech, USA). The total RNA isolation was performed in replicates.

### Complementary DNA (cDNA) synthesis and quantitative real-time (qRT) PCR analysis

Total RNA was quantified at 260 and 280 nm wavelengths with the help of a ND-2000 UV spectrophotometer (Nanodrop Technologies, Wilmington, DE, USA). Synthesis of cDNA was carried out with 5 μg of total RNA by using Verso cDNA synthesis kit (Thermo scientific, USA) as per the manufacturer’s instructions. The quantification of cDNA was performed to obtain equal concentration (100 ng) with the help of ND-2000 UV spectrophotometer. A total of 25 selected genes from glycolysis, serine biosynthesis and diterpene alkaloid biosynthesis were subjected to qRT-PCR analysis. The primers of the selected genes were designed from transcriptomic sequences of *A. heterophyllum* (Pal et al. 2015) by using Primer3 software (http://bioinfo.ut.ee/primer3-0.4.0/). The details of the primers with annealing temperatures are provided in supplementary Table 1. The expression analysis of selected genes was conducted in quadruplicates on CFX96 system (Bio-Rad Laboratories; Hercules, CA, USA) as described in Kumar et al. (2015d). The reference genes, 26S and GAPDH were used as the standard genes in this study.

### Preparation of plant extract and quantification of steviol

*A. heterophyllum* roots of high (AHSR) and low (AHKR) content accessions were homogenized in a prechilled pestle and mortar using liquid nitrogen. Steviol was extracted from 1 g of each powdered sample with 10 mL of 50 % ethanol. The samples were then sonicated for 30 min at 30 °C and passed through 0.22 μm filter (Millipore). To 0.01 mL of each filtered sample, 0.99 mL of 50 % methanol was added to make 100-fold dilution and 20 μL injection of each diluted sample was used for the quantification of steviol. The quantification of steviol was carried by using RP- HPLC.

(Waters 515) through C18 (5 µm) 4.6 × 250 mm Waters Symmetry Column using PDA detector (Waters 2996). The mobile phase was generated by a gradient elution programme using two solvent systems, i.e., Solvent A (0.1 % trifluoroacetic acid in Milli-Q water) and Solvent B (acetonitrile). Gradient elution started at isocratic 5 % B for 5 min, increased to 95 % B over 20 min, returned to 5 % B for 5 min and held at 95 % B for 5 min with a flow rate of 1 mL/min. Detection of steviol was carried out at an absorbance of 200 ± 4 nm wavelength. The cycle time of analysis was 30 min at 50 °C. The compound was identified on the basis of retention time and comparison of UV spectra with the steviol standard (Chromadex, USA). The experiment was performed in triplicates.

### Statistical analysis

The quantitative gene expression analysis was obtained by calculating the mean ± SD from quadruplicates. The statistical analysis was done by using two-way ANOVA followed by a Bonferroni test using GraphPad prism software version 6.0. Pearson’s correlation coefficients were calculated by using Excel (Microsoft, USA) and significant levels were tested at *P* < 0.05 using the Students *t* test.

## Results

### Atisine biosynthetic pathway

Here, we proposed that atisine in *A. heterophyllum* might have originated from *ent*-kaurene and *ent*-atisane diterpenoid alkaloid families via the formation of steviol and atisenol intermediates. The *ent*-kaurene and *ent*-atisane DAs are thought to be biosynthesized from C_20_ diterpene precursor GGPP (Zhao et al. 2009). GGPP is cyclized enzymatically to give *ent*-CPP which is a key branch point in the reaction pathways leading to *ent*-kaurene and *ent*-atisane-type DAs (Devkota and Sewald 2013). The *ent*-kaurene is the intermediate in biosynthesis of gibberellin and is converted into steviol via formation of kaurenol, kaurenal and kaurenoic acid (Humphrey et al. 2006; Kelling et al. 2010). Recently, steviol was successfully used as an intermediate to access atisine in vitro (Cherney et al. 2015). On the other hand, atisenol has been earlier isolated from *A. heterophyllum* by Pelletier et al. (1982). Compared with the structure of atisine, the steviol and atisenol lack one unit of ethanolamine. The later one is biosynthesized from decarboxylation of serine in plants (Rontein et al. 2003). According to the reports, ethanolamine or l-serine could be the possible nitrogen source involved in the biosynthesis of atisine-type DAs (Zhao et al. 2009). Further existence of multiple pathways for serine biosynthesis in plants, viz. phosphorylated, glycolate and glycerate pathways complicated our realization of atisine production in *A. heterophyllum* (Ros et al. 2014). Moreover, these pathways remain silent in the absence of specific trigger from primary metabolism. Therefore, we have proposed for the first time the complete atisine biosynthetic pathway which showed the integration of glycolysis, MVA/MEP, serine biosynthesis and diterpene biosynthesis into atisine in *A. heterophyllum* (Fig. 1).

### Detection of intermediates in atisine biosynthetic pathway

**Fig. 2:**
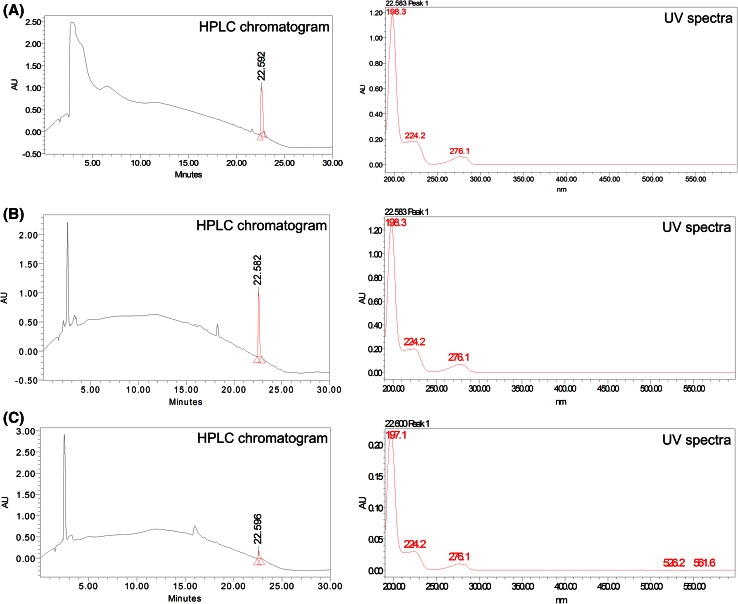
HPLC analysis of steviol quantification in *A. heterophyllum* accessions. **a** HPLC chromatogram and UV spectra of steviol standard, **b** HPLC chromatogram and UV spectra of high content accession, **c** HPLC chromatogram and UV spectra of low content accession

Atisine was not detected in the shoots of *A. heterophyllum* plants (Malhotra et al. 2014). In contrast, the level of atisine in roots of high content accession was 0.37 %, which was 3-fold (*p* < 0.001) greater compared to that in roots of low content accession (0.14 %) (Malhotra et al. 2014). Further, this study showed that the level of steviol in roots of high content accession was 0.06 %, which was 6.0-fold (*p* < 0.001) greater as compared to that in roots of low content accession (0.01 %). The HPLC chromatograms and UV spectra of the steviol standard and the samples are provided in Fig. 2. It was evident from the results that steviol could be involved in the biosynthesis of atisine as latter has been found to be biosynthesized and accumulated exclusively in roots of *A. heterophyllum*.

### Mapping genes to enzymatic steps and shortlisting particular paralogs

The mining of transcriptomes for individual gene sequences is a cumbersome process; therefore, KEGG automatic annotation server (KAAS) was used for mapping each gene to enzymatic steps as described in Pal et al. (2015). The genes corresponding to enzymatic steps were successfully mapped and shortlisted paralogs are shown in supplementary Tables 2 and 3.

### Expression status of pathways genes vis-à-vis atisine content

Total RNA samples were prepared from *A. heterophyllum* shoots and roots of high content accession grown under controlled conditions at greenhouse of Jaypee University of Information Technology. The cDNA was prepared and subjected to quantitative RT-PCR analysis which was performed for 25 genes belonging to glycolysis, phosphorylated, glycolate, glycerate and diterpene biosynthetic pathways in all the samples. The relative delta CT values obtained from pre-processing the raw qPCR data were used to calculate the gene expression values.

**Fig. 3:**
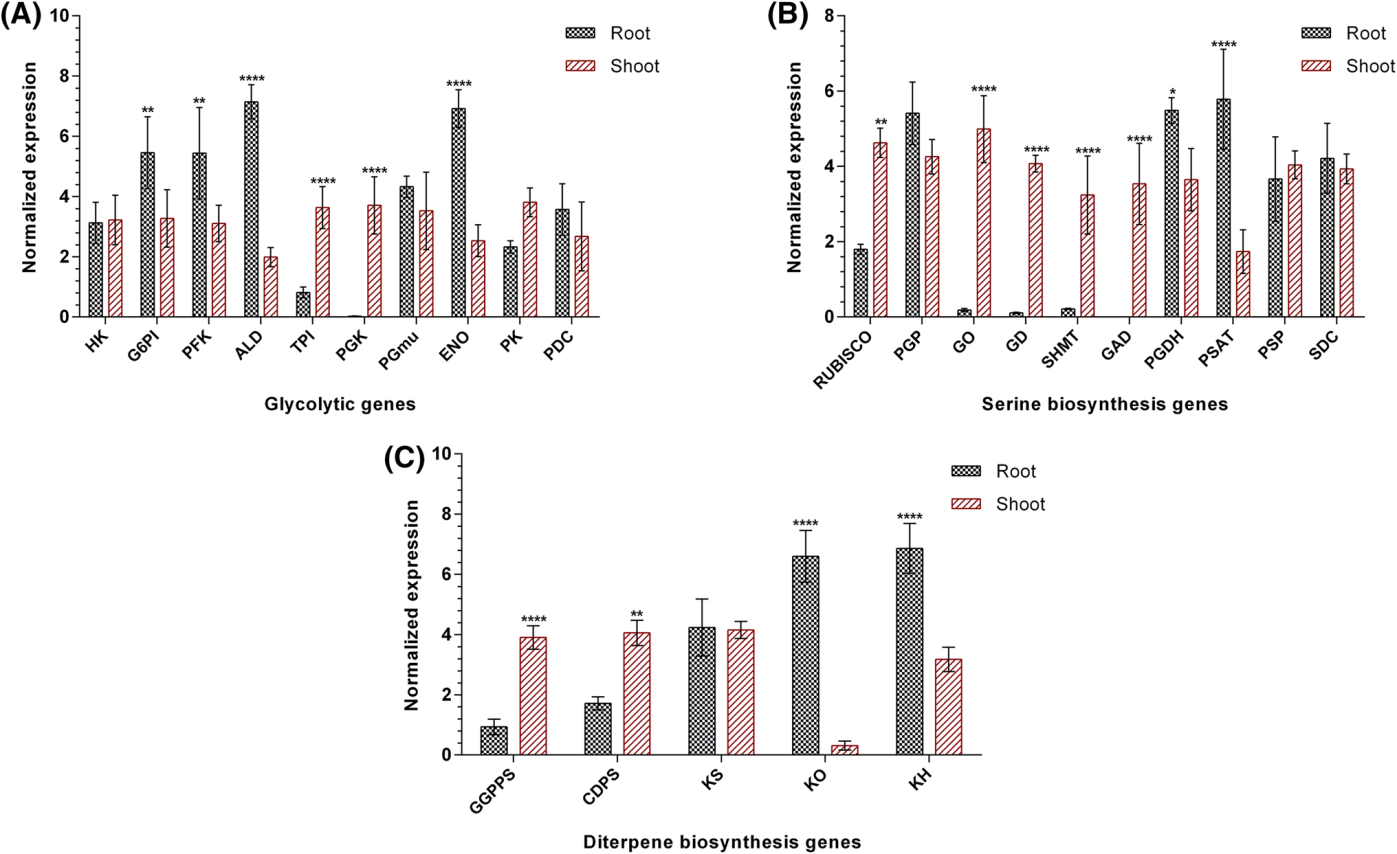
Quantitative expression analysis of selected genes in different tissues of high content accession of *A. heterophyllum*; **a** glycolysis, **b** serine biosynthesis and, **c** diterpene biosynthesis. The vertical axis represents the normalized expression and horizontal axis represents the different genes. Expression level was normalized to housekeeping genes, i.e., 26S and GAPDH. Bar graphs show mean ± SD (*n* = 4). Significance was evaluated for each gene between different tissues (*p < 0.05, **p < 0.01, ****p < 0.0001). Abbreviations are elaborated in supplementary Table 4

The expression of different genes belonging to integrated pathways showed significant modulations in congruence with atisine content between root and shoot tissues of *A. heterophyllum*. Among the glycolytic genes, 4 genes exhibited up-regulation, 2 genes were down-regulated while 4 genes showed non-significant modulation in roots compared to shoots (Fig. 3a). The G6PI, PFK, ALD and ENO genes were up-regulated by 1.67- (*p* < 0.01), 1.75- (*p* < 0.01), 3.60- (*p* < 0.0001) and 2.73-fold (*p* < 0.0001), respectively, whereas the expression of TPI and PGK was down-regulated by 0.22- (*p* < 0.0001) and 0.01-fold (*p* < 0.0001), respectively. Further, 2 genes corresponding to phosphorylated pathway of serine biosynthesis, i.e., PGDH and PSAT exhibited 1.50- (*p* < 0.05) and 3.33-fold (*p* < 0.0001) up-regulation, respectively, while transcript encoding PSP enzyme showed non-significant modulation in roots compared to shoots. Conversely, 3 genes of glycolate pathway, i.e., GO, GD and SHMT showed significant down-regulation with 0.04- (*p* < 0.0001), 0.03- (*p* < 0.0001) and 0.07-fold (*p* < 0.0001), respectively, while one gene, i.e., PGP showed non-significant modulation in roots compared to shoots (Fig. 3b). On the other hand, GAD of glycerate pathway showed significant expression only in shoots (3.53, *p* < 0.0001) of *A. heterophyllum*. The transcript encoding SDC showed non-significant change in expression level between root and shoot tissues.

Among the diterpene biosynthetic genes, 2 genes exhibited up-regulation, 2 genes were down-regulated while 1 gene (KS) showed non-significant modulation in roots compared to shoots (Fig. 3c). The KO and KH genes were up-regulated by 21.29- (*p* < 0.0001) and 2.16-fold (*p* < 0.0001), respectively, whereas the expression of GGPPS and CDPS was down-regulated by 0.24- (*p* < 0.0001) and 0.42-fold (*p* < 0.01), respectively.

It was evident from the results that G6PI, PFK, ALD, ENO, PGDH, PSAT, KO and KH genes significantly correlated with atisine content between root and shoot tissues of *A. heterophyllum*, thereby indicating their involvement in atisine biosynthesis.

**Fig. 4:**
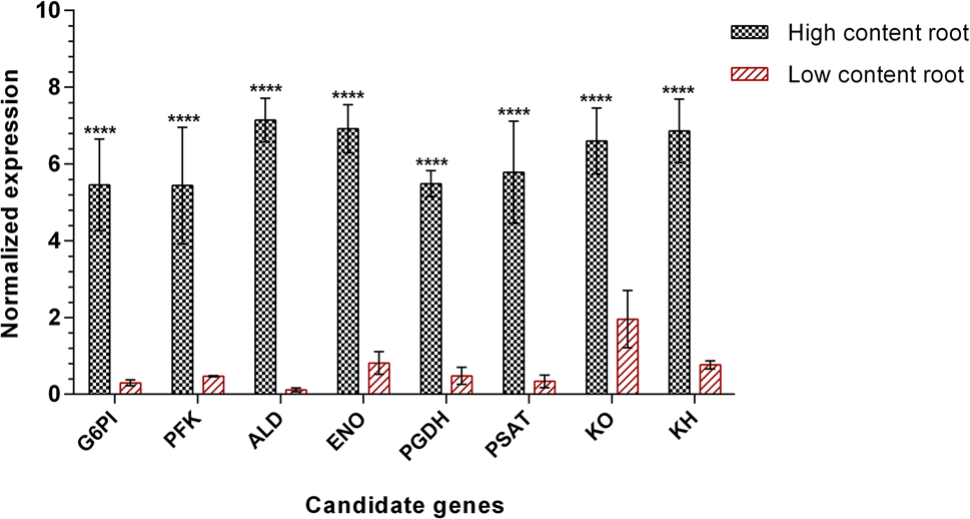
Quantitative expression analysis of shortlisted genes in roots of high *vs* low content accessions of *A. heterophyllum*. The vertical axis represents the normalized expression and horizontal axis represents the different genes. Expression level was normalized to housekeeping genes, i.e., 26S and GAPDH. *Bar graphs* show mean ± SD (*n* = 4). Significance was evaluated for each gene between different accessions (****p < 0.0001). Abbreviations are elaborated in supplementary Table 4

It is likely that the higher expression observed for these genes in initial screening might not only be due to variation in atisine content but also attributed to different tissues and differential production of other secondary metabolites. Therefore, to further ascertain the involvement of selected transcripts in atisine biosynthesis, the expression levels were checked on roots of high content accession (AHSR) and low content accession (AHKR) varying in atisine content with 0.37 and 0.14 %, respectively. This analyses revealed a striking increase in transcript level of G6PI, PFK, ALD, ENO, PGDH, PSAT, KO and KH enzymes in roots of high content accession to the tune of 18.26- (*p* < 0.0001), 11.54- (*p* < 0.0001), 62.36- (*p* < 0.0001), 8.51- (*p* < 0.0001), 11.44- (*p* < 0.0001), 17.15- (*p* < 0.0001), 3.37- (*p* < 0.0001) and 8.97-fold (*p* < 0.0001), respectively, compared to low content accession (Fig. 4). This shortlisting, thus, reflected the involvement of selected genes in the regulation of atisine biosynthesis in *A. heterophyllum*.

## Discussion

The goal of present study was to fill a gap in our knowledge of atisine biosynthesis and encoded genes. We have used the bio-retrobiosynthetic approach for formal interpretation of atisine biosynthesis pattern and integrated molecular layers to tap the detailed role of genes related to different feeder pathways for atisine biosynthesis in *A. heterophyllum*. Atisine biosynthesis involved seven interlinking metabolic processes, including glycolysis, phosphorylated, glycolate, glycerate, mevalonate, non-mevalonate and diterpene biosynthetic pathways. It is, however, clear that the dovetails of the genes to atisine biosynthesis remain murky.

**Fig. 5:**
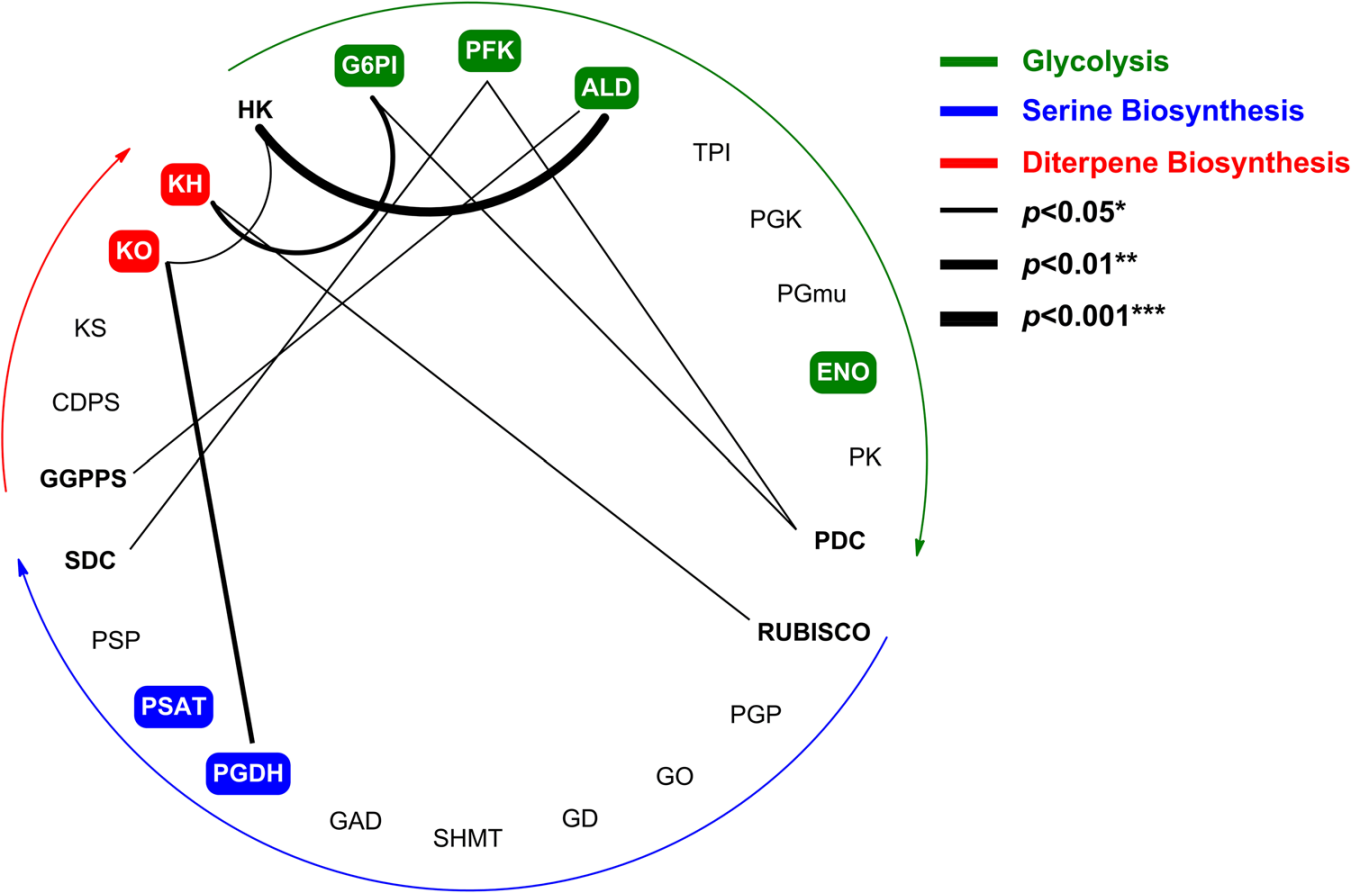
Positively co-expressed network of shortlisted genes during atisine biosynthesis in roots of *A. heterophyllum*. In each metabolic pathway, shortlisted genes are boxed with different color and significant positive correlations between the gene expression levels are shown by connected lines. Abbreviations are elaborated in supplementary Table 4

This study has revealed the complete biosynthetic pathway of atisine along with candidate genes in *A. heterophyllum* for the first time. The expression analysis results of all the 25 selected genes showed modulated expression vis-à-vis atisine content in root and shoot tissues of *A. heterophyllum*. The atisine content showed 0–0.37 % increase in root compared to shoot tissue. The higher expression level of genes encoding G6PI (1.67-fold), PFK (1.75-fold), ALD (3.60-fold) and ENO (2.73-fold) enzymes of glycolysis in roots might indicate that these genes contribute to atisine biosynthesis by producing higher levels of precursors for connecting pathways, viz. mevalonate, non-mevalonate (MEP) and serine biosynthetic pathways leading to their activation (Kumar et al. 2015c; Munoz-Bertomeu et al. 2013). This was also supported by correlation analyses which showed significant positive correlations (*p* < 0.05; *p* < 0.01; *p* < 0.001) between shortlisted transcripts of glycolysis, serine biosynthesis and diterpene biosynthesis (Fig. 5). The combined effect of increased expression levels of G6PI, PFK and ALD would produce higher levels of glyceraldehyde-3-phosphate, the substrate for DXPS and GAPDH enzymes (Mutuku and Nose 2012). DXPS, which catalyzes the rate limiting step in MEP pathway, could produce higher levels of pathway intermediates resulting in the activation of MEP pathway (Broun and Somerville 2001). Malhotra et al. (2014) also showed the activation of MEP pathway in root tissue of *A. heterophyllum* known to produce higher levels of atisine. Stimulation of MEP pathway might be linked with the increased supply of GGPP, which is a precursor for the diterpene biosynthesis (Malhotra et al. 2014; Zhao et al. 2009). GAPDH, on the other hand, serves as the housekeeping gene and did not show significant alteration in expression in root and shoot tissues of *A. heterophyllum*. This might be indicative of glycolysis homeostasis irrespective of its flux towards non-mevalonate pathway. The expression of ENO gene showed noticeable increase (2.73-fold) in root over shoot tissue which might produce higher levels of PEP, the substrate for the enzyme DAHPS and PK. The supply of PEP is limiting for the shikimate pathway. Voll et al. (2009) observed that antisense inhibition of ENO enzyme hampered the plastidic shikimate pathway. It has been reported that shikimate/phenylpropanoid pathway is supposed to be the entry point into alkaloid biosynthesis via the formation of tyrosine (Tzin and Galili 2010). Our observations are also in agreement with another study which showed that total alkaloid content is higher in roots of high content accessions compared to low content accessions of *A. heterophyllum* (Malhotra et al. 2014). The expression of PK gene showed no significant modulation in root and shoot tissue which might indicate that higher concentrations of PEP is likely to be associated with enhanced allocation into shikimate/phenylpropanoid pathway but might also not affect the pyruvate production. This was further supported by correlation analysis results which represented that no significant positive correlation of ENO gene was found with other studied genes (Fig. 5). This observation implied that enhanced expression of ENO gene might be correlated with elevated total alkaloid biosynthesis in high content accessions but not affecting the biosynthesis of atisine through pyruvate homeostasis.

Further, serine biosynthesis is sensed via three pathways, including one that is related to photorespiration (glycolate pathway), others are nonphotorespiratory pathways but one requires phosphorylation (phosphorylated pathway), and one does not require phosphorylation (glycerate pathway) (Ros et al. 2014). It is thus tempting to identify the consensus route for serine production in *A. heterophyllum* roots. Our results indicated that only the genes encoding PGDH and PSAT enzymes in the phosphorylated pathway showed significant elevation in expression levels in root compared to shoot tissues. This might indicate that phosphorylated pathway plays a pivotal role inevitably over other pathways for serine production in root tissues of *A. heterophyllum*. Cascales-Minana et al. (2013) also showed that phosphorylated pathway is essential for root growth and has a major role in l-serine biosynthesis in non-photosynthetic tissues. Moreover, we also found highly significant positive correlation (*p* < 0.01) between PGDH of phosphorylated pathway and KO of diterpene biosynthetic pathway genes during their concomitant biosynthesis suggesting two parallel pathways for the production of atisine.

Interestingly, the demonstration of diterpene biosynthetic genes also revealed higher expression level of two genes, viz. KO (21.29-fold) and KH (2.16-fold) in roots which provide support to the involvement of steviol in the biosynthesis of atisine in our proposed atisine biosynthetic pathway. The role of KO and KH genes in atisine biosynthesis was further ascertained through their elevated expression levels in roots of high content accessions compared to low content accessions. This observation was in agreement with further evidence and supportive data of steviol quantification which showed 6.0-fold increase in roots of high content accessions compared to low content accessions. Surprisingly, we did not find any upstream gene of diterpene biosynthesis whose expression level is in congruence with atisine content. This might be theoretically explained by the fact that atisine biosynthesis bifurcated after *ent*-CPP formation and no information is available as of today for the enzymes involved in the biosynthesis of atisine via atisenol route (Devkota and Sewald 2013). However, this might indicate that these genes may serve as the core factors in the control of atisine biosynthesis but unambiguously proved after the elucidation of unknown genes in the atisine biosynthetic pathway.

Taken together, the correlation approach used in the present study not only reinforced plausible atisine biosynthetic pathway but also provided a refined list of candidate genes which might regulate the atisine biosynthesis in *A. heterophyllum*. For multistep pathways, contemplated molecular switches are major undertakings which entails the metabolic engineering of pathways suffice to meet the increasing demands of desired secondary metabolites in target plant species. This study, thus, provides a platform for designing a suitable genetic intervention strategy to elevate the production of atisine in *A. heterophyllum* in the near future.

## Conclusions

The complete biosynthetic pathway of atisine has been elucidated for the first time in *A. heterophyllum*. This work highlights the candidate genes in glycolysis (G6PI, PFK, ALD and ENO), serine biosynthesis (PGDH and PSAT) and diterpene biosynthesis (KO and KH) and showed that phosphorylated pathway is a major contributor of serine for atisine production. The quantification of steviol in roots of high *vs* low content accessions revealed that atisine biosynthesis is regulated by two core modules, viz. atisenol and steviol in *A. heterophyllum*. This study provides a snapshot of atisine biosynthesis and associated bottlenecks in *A. heterophyllum* but actual realization necessitates a next level investigation to get a robust overview of exact mechanism behind atisine biosynthesis by elucidating missing enzymes and gene function analysis.

## Electronic supplementary material

Below is the link to the electronic supplementary material.

Supplementary material 1 (DOC 173 kb)
