# Supplementary material for: Molecular dissection of pathway components unravel atisine biosynthesis in a non-toxic Aconitum species, A. heterophyllum Wall
Source: 3 Biotech. 2016 Apr 18;6(1):106. doi: 10.1007/s13205-016-0417-7 (PMC4835424; doi:10.1007/s13205-016-0417-7)
Supplement: Supplementary file 1 — Supplementary material 1 (DOC 173 kb) [file 13205_2016_417_MOESM1_ESM.doc]

**Supplementary Table 1. Primer sequences of selected genes used in quantitative RT-PCR analysis**

| **Genes** | **Primer sequence** | **Fragment size (bp)** | **Annealing temperatures (^0^C)** |
| --- | --- | --- | --- |
| 26S | FP 5’-CACAATGATAGGAAGAGCCGAC-3’  RP 5’-CAAGGGAACGGGCTTGGCAGAATC-3’ | 500 | 58 |
| HK | FP 5’-CATCCATGGCTATTACAGTC-3’  RP 5’-AAAACCAGAAAGGTAGTGGT-3’ | 151 | 51 |
| G6PI | FP 5’-CTAACCTGTTGGATATGTGG-3’  RP 5’-TACCTGTGCTTCTAGGTTTG-3’ | 121 | 51 |
| PFK | FP 5’-CCGAGACACAAAATAAGGTA-3’  RP 5’-TACTTCTTCCCTCTGATCCT-3’ | 119 | 51 |
| ALD | FP 5’-GAAAACATAGAAGCTGGACA-3’  RP 5’-TCAGTAGGTGTATCCCTTGA-3’ | 138 | 51 |
| TPI | FP 5’-GGTGACATTATCAAATCTGC-3’  RP 5’-GTTGCTGAACAAGAAAGGTA-3’ | 146 | 50 |
| GAPDH | FP 5’-TACCCAGTAAACTTTGTTGC-3’  RP 5’-ACTTCAAGGGAGAACAATCT-3’ | 140 | 51 |
| PGK | FP 5’-TGTTATCTGGAATGGACCTA-3’  RP 5’-CACCTCCAATTATTGTTGTC-3’ | 122 | 50 |
| PGmu | FP 5’-GATAGGGTTGTTGAGTGATG-3’  RP 5’-CGAAACCTATACTTGAACCA-3’ | 152 | 53 |
| ENO | FP 5’-ATCAAGCTGTTGTACCATGT-3’  RP 5’-GGTGTATCAAAGGCTGTAGA-3’ | 111 | 53 |
| PK | FP 5’-AACAGACTCATTTGACTGGA-3’  RP 5’-GGGCAGCTTCTAATATCTCT-3’ | 143 | 50 |
| PDC | FP 5’-CCTAAGCTCCTGAGAGAAAT-3’  RP 5’-TGCTTGTAGTGAAAACCTTC-3’ | 121 | 51 |
| RUBISCO | FP 5’-GCTTCTTCAATGATGTTGTC-3’  RP 5’- GGGAGAGATGTAAGGTCATT-3’ | 146 | 54 |
| PGP | FP 5’- TGTGATATGGAAGGGAGATA-3’  RP 5’- AGAAACTTGTTTGCGTACTG-3’ | 135 | 51 |
| GO | FP 5’- ACAAGGACAGGAAGGTAGTT-3’  RP 5’- TCTTCAGAGTCAAATGTGGT-3’ | 153 | 53 |
| GD | FP 5’- TCGACATCATCTAAAGTGGT-3’  RP 5’- TTCCTTTCTTCGATACTGTG-3’ | 148 | 50 |
| SHMT | FP 5’- AACTCATACCTTCCGAGAAT-3’  RP 5’- TTCTGACATAAGGTTTCTGC-3’ | 148 | 50 |
| GAD | FP 5’- GGTCAGTTGACAGAAGATTG-3’  RP 5’- AGCAACTCCACACTTGTTAG-3’ | 132 | 50 |
| PGDH | FP 5’- TACACGCTCCTCGATTATAC-3’  RP 5’- GTGACCTAGACACGAGACTG-3’ | 137 | 52 |
| PSAT | FP 5’- CTCAGACTTCTCCAAAGTGA-3’  RP 5’- GCAGGTTGAGAAGAACAATA-3’ | 139 | 51 |
| PSP | FP 5’- CAGCATCTCGAAGTTGTACT-3’  RP 5’- GGAAGGTTAAGGGATACAAG-3’ | 123 | 53 |
| SDC | FP 5’- AATCCTTGGGTAAGGATAAC-3’  RP 5’- TCAATGAACCAGAGACTCAT-3’ | 126 | 51 |
| GGPPS | FP 5’- GACCAGTCATAAGGTGTTTG-3’  RP 5’- CTATCGCCATTATCATCCTA-3’ | 128 | 50 |
| CDPS | FP 5’- GCTTAATCTTCTCCATGTCA-3’  RP 5’- TGTAGGGATACCTGCTAATG-3’ | 100 | 47 |
| KS | FP 5’- TCTATTTTGGCGTAGTTGAC-3’  RP 5’- GAGATGTCGCTCATAGTGTC-3’ | 119 | 49 |
| KO | FP 5’- GGAGAAGAGCAGTGTGTAAA-3’  RP 5’- TCATCCTCTGTGAACTGTTT-3’ | 124 | 52 |
| KH | FP 5’- TGCTGAAGGACTTAATCAAC-3’  RP 5’- TCCTCCAGGAAGAGTAAGAT-3’ | 136 | 53 |

**Supplementary Table 2. Information of functionally characterized sequences from other plant species used for the selection of correct paralogs of studied genes.**

| **S. No.** | **Name** | **No. of entries in transcriptome** | |  | **Functionally characterized sequences submitted in NCBI/TAIR/UniProt** | | | | | |
| --- | --- | --- | --- | --- | --- | --- | --- | --- | --- | --- |
|  |  | **Roots** | **Shoots** | **Accession No.**  **nucleotide** | **Seq. length (bp)** | **Sequence** | **Accession No.**  **protein** | **Seq. length (a.a)** | **Source** | **Reference** |
| 1 | Hexokinase | 8 | 6 | AF196966  DQ835563 | 2097  1497 | Complete cds  Complete cds | AAG28503  ABI18156 | 498  498 | *Citrus sinensis*  *Helianthus annuus* | (Li et al. 2003)  (Troncoso-Ponce et al. 2011) |
| 2 | G-6-P I | 9 | 3 | U17225  FJ210719 | 2051  2306 | Complete cds  Complete cds | AAA82734  ACI29026 | 567  659 | *Zea mays*  *Dunaliella salina* | (Lal & Sachs 1995)  (Cui et al. 2010) |
| 3 | PFK | 6 | 1 | DQ437576 | 1906 | Complete cds | ABD96051 | 499 | *Spinacia oleracea* | (Winkler et al. 2007) |
| 4 | Aldolase | 18 | 7 | M97476  EU975551 | 1227  1387 | 3’-end cds  Complete cds | Q01516  ACG47669 | 356  396 | *Pisum sativum*  *Zea mays* | (Razdan et al. 1992)  (Alexandrov et al. 2009) |
| 5 | TPI | 3 | 1 | S70730  AY438596 | 1064  1079 | -  Complete cds | AAB30759  AAR11379 | 257  254 | *Stellaria longipes*  *Solanum chacoense* | (Zhang & Chinnappa 1994)  (Dorion et al. 2005) |
| 6 | GAPDH | 7 | 7 | DQ173770 | 1349 | Complete cds | ABA03227 | 341 | *Populus maximowiczii × Populus nigra* | (Page et al. 2007) |
| 7 | PGK | 5 | 5 | HQ450764 | 1722 | Complete cds | ADR71054 | 481 | *Nicotiana benthamiana* | (Cheng et al. 2013) |
| 8 | PGmu | 16 | 11 | AF047842 | 1940 | Complete cds | AAD24857 | 559 | *Solanum tuberosum* | (Westram et al. 2002) |
| 9 | Enolase | 8 | 3 | FJ979826  AY307448 | 1335  1580 | Complete cds  Complete cds | ACR56690  AAQ77240 | 444  444 | *Nicotiana tabacum*  *Brassica rapa* | (Voll et al. 2009)  (Zhao et al. 2004) |
| 10 | PK | 26 | 17 | DQ114474 | 1824 | Complete cds | AAZ86534 | 511 | *Capsicum annuum* | (Kim et al. 2006) |
| 11 | RUBISCO | 10 | 10 | JX576219  L24073 | 546  1355 | Complete cds  Complete cds | AFW90575  AAB02583 | 181  401 | *Solanum tuberosum*  *Oryza sativa* | (Gangadhar et al. 2014)  (Zhang et al. 1995) |
| 12 | PGP | 3 | 3 | JQ717275  NM_124150  EU976812 | 125  1203  1233 | Partial cds  Complete cds  Complete cds | AFP89348  NP_199587  ACG48930 | 41  301  273 | *Prunus persica*  *Arabidopsis thaliana*  *Zea mays* | (Zhou et al. 2013)  (Zhou et al. 2013)  (Alexandrov et al. 2009) |
| 13 | GO | 2 | 8 | HQ110098  J03492 | 1116  1511 | Complete cds  Complete cds | ADM26718  AAA34030 | 371  369 | *Nicotiana benthamiana*  *Spinacia oleracea* | (Rojas et al. 2012)  (Volokita & Somerville 1987) |
| 14 | GAD | 1 | 3 | - | - | - | - | - | - | - |
| 15 | PGDH | 3 | 4 | AT1G17745 | 1956 | Full length cds | AT1G17745 | 651 | *Arabidopsis thaliana* | (Toujani et al. 2013) |
| 16 | PSAT | 1 | 1 | EU969783 | 1632 | Complete cds | ACG41901 | 425 | *Zea mays* | (Alexandrov et al. 2009) |
| 17 | PSP | 1 | 0 | AT1G18640 | 888 | Full length cds | AT1G18640 | 295 | *Arabidopsis thaliana* | (Cascales-Minana et al. 2013) |
| 18 | GD | 1 | 0 | - | - | - | - | - | - | - |
| 19 | SHMT | 13 | 7 | M87649 | 1803 | Complete cds | AAA33687 | 518 | *Pisum sativum* | (Turner et al. 1992) |
| 20 | PDC | 13 | 4 | AF195868  DQ001726  AY263388 | 1930  2048  2010 | Partial cds  Complete cds  Complete cds | AAG22488  AAZ05069  AAP96920 | 575  589  605 | *Vitis* v*inifera*  *Citrus sinensis*  *Dianthus caryophyllus* | (Or et al. 2000)  (Pasentsis et al. 2007)  (Owen et al. 2004) |
| 21 | GGPPS | 4 | 6 | AB205047 | 1305 | Complete cds | BAE79550 | 345 | *Chrysanthemum morifolium* | (Kishimoto & Ohmiya 2006) |
| 22 | CDPS | 11 | 4 | AF034545 | 2590 | Complete cds | AAB87091 | 787 | *Stevia rebaundiana* | (Richman et al. 1999) |
| 23 | KS | 2 | 6 | AF097310 | 3117 | Complete cds | AAD34294 | 784 | *Stevia rebaundiana* | (Richman et al. 1999) |
| 24 | KO | 42 | 15 | AY364317 | 1645 | Complete cds | AAQ63464 | 513 | *Stevia rebaundiana* | (Humphrey et al. 2006) |
| 25 | SDC | 0 | 2 | AF389349 | 1584 | Complete cds | AAK77493 | 482 | *Arabidopsis thaliana* | (Rontein et al. 2001) |

**Supplementary Table 3. Shortlisting of correct paralogs for studied genes by comparison with functionally characterized sequences of other plant species and transcripts abundance.**

| **S. No.** | **Name** | **blastn^a^/tblastn^b^ (with characterized sequences as query and transcripts of both roots and shoots as subject sequences)** | | | **Selected transcript ID** | | **Sequence length** | | **FPKM (fragments per kilobase per million sequenced reads) value** | |
| --- | --- | --- | --- | --- | --- | --- | --- | --- | --- | --- |
|  |  | **E-value** | **Query coverage (%)** | **% homology** | **Roots** | **Shoots** | **Roots** | **Shoots** | **Roots** | **Shoots** |
| 1 | Hexokinase | 0.0^a^, 0.0^b^  0.0^a^, 0.0^b^ | 71^a^, 94^b^  98^a^, 94^b^ | 77^a^, 81^b^  74^a^, 76^b^ | 75440  75440 | 3959  3959 | 1775  1775 | 2084  2084 | 32.66  32.66 | 83.27  83.27 |
| 2 | G-6-P I | 0.0^a^, 0.0^b^  3e-124^a^, 0.0^b^ | 79^a^, 100^b^  52^a^, 84^b^ | 75^a^, 76^b^  69^a^, 69^b^ | -  - | 21032  21032 | -  - | 1786  1786 | -  - | 33.74  33.74 |
| 3 | PFK | 0.0^a^, 0.0^b^ | 68^a^, 87^b^ | 74^a^, 77^b^ | 12746 | - | 1787 | - | 13.3 | - |
| 4 | Aldolase | 0.0^a^, 0.0^b^  0.0^a^, 0.0^b^ | 87^a^, 99^b^  75^a^, 97^b^ | 79^a^, 89^b^  77^a^, 82^b^ | 11739  2609 | -  - | 1504  1589 | -  - | 28.81  457.45 | -  - |
| 5 | TPI | 9e-70^a^, 1e-119^b^  0.0^a^, 1e-151^b^ | 68^a^, 98^b^  69^a^, 100^b^ | 69^a^, 67^b^  80^a^, 80^b^ | 26505  26505 | -  - | 1018  1018 | -  - | 235.32  235.32 | -  - |
| 6 | GAPDH | 0.0^a^, 2e-169^b^ | 74^a^, 99^b^ | 81^a^, 83^b^ | - | 4817, 4818 | - | 1493, 1718 | - | 790.01, 38.87 |
| 7 | PGK | 0.0^a^, 0.0^b^ | 71^a^, 98^b^ | 81^a^, 85^b^ | 11940 | - | 1848 | - | 28.5 | - |
| 8 | PGmu | 0.0^a^, 0.0^b^ | 86^a^, 100^b^ | 78^a^, 79^b^ | - | 3761 | - | 1958 | - | 92.4 |
| 9 | Enolase | 0.0^a^, 0.0^b^  0.0^a^, 0.0^b^ | 93^a^, 93^b^  79^a^, 93^b^ | 80^a^, 88^b^  79^a^, 90^b^ | 1268  1268 | -  - | 1475  1475 | -  - | 386.17  386.17 | -  - |
| 10 | PK | 0.0^a^, 0.0^b^ | 84^a^, 100^b^ | 78^a^, 87^b^ | 643, 645, 646, 647 | 10511, 10512, 10513 | 2483, 2223, 2212, 2657 | 2126, 1958, 2146 | 0.87, 1.99, 4.55, 83.5 | 3.95, 6.44, 34.7 |
| 11 | RUBISCO | 4e-94^a^, 2e-91^b^  0.18^a^, 7e-04^b^ | 87^a^, 87^b^  1^a^, 11^b^ | 76^a^, 73^b^  100^a^, 31^b^ | 25515  - | -  17236 | 738  - | -  237 | 4.53  - | -  5.35 |
| 12 | PGP | 3e-22^a^, 7e-22^b^  2e-138^a^, 1e-173^b^  -, 5e-05^b^ | 76^a^, 90^b^  63^a^, 98^b^  -, 32^b^ | 80^a^, 89^b^  74^a^, 75^b^  -, 25^b^ | -  21112 | 4414  -  4414 | -  1244  - | 1092  -  1092 | -  17.89  - | 17.52  -  17.52 |
| 13 | GO | 0.0^a^, 0.0^b^  0.0^a^, 0.0^b^ | 95^a^, 95^b^  70^a^, 100^b^ | 78^a^, 86^b^  79^a^, 83^b^ | 36604  - | -  7954 | 1447  - | -  1528 | 12.42  - | -  181.84 |
| 14 | GAD | - | - | - | - | - | - | - | - | - |
| 15 | PGDH | 0.0^a^, 0.0^b^ | 82^a^, 87^b^ | 72^a^, 69^b^ | - | 26560 | - | 2233 | - | 38.49 |
| 16 | PSAT | 1e-56^a^, 0.0^b^ | 45^a^, 86^b^ | 67^a^, 71^b^ | 3589 | 8756 | 1710 | 1586 | 96.28 | 14.72 |
| 17 | PSP | 4e-123^a^, 2e-147^b^ | 79^a^, 100^b^ | 74^a^, 68^b^ | 10924 | - | 1311 | - | 13.36 | - |
| 18 | GD | - | - | - | 464* | - | 1425 | - | 5.03 | - |
| 19 | SHMT | 0.0^a^, 0.0^b^  0.0^a^, 0.0^b^ | 88^a^, 99^b^  88^a^, 99^b^ | 80^a^, 89^b^  81^a^, 87^b^ | -  1226 | 14255  - | -  1868 | 1861  - | -  128.77 | 121.4  - |
| 20 | PDC | 0.0^a^, 0.0^b^  0.0^a^, 0.0^b^  0.0^a^, 0.0^b^ | 86^a^, 98^b^  83^a^, 96^b^  83^a^, 92^b^ | 74^a^, 77^b^  79^a^, 86^b^  72^a^, 79^b^ | -  -  - | 26982  26982  26982 | -  -  - | 2122  2122  2122 | -  -  - | 40.22  40.22  40.22 |
| 21 | GGPPS | 2e-115^a^, 1e-165^b^ | 69^a^, 91^b^ | 70^a^, 72^b^ | 15106 | - | 1412 | - | 16.02 | - |
| 22 | CDPS | 2e-104^a^, 0.0^b^ | 48^a^, 89^b^ | 67^a^, 50^b^ | 132 | - | 2636 | - | 4.84 | - |
| 23 | KS | 2e-05^a^, 2e-79^b^ | 4^a^, 62^b^ | 87^a^, 33^b^ | 135 | - | 2712 | - | 3.02 | - |
| 24 | KO | 1e-127^a^, 0.0^b^ | 81^a^, 92^b^ | 68^a^, 57^b^ | 4803, 4806 | - | 1923, 2753 | - | 178.77, 5.52 | - |
| 25 | SDC | - | - | - | - | - | - | - | - | - |

^a^ Homology identity of nucleotides

^b^ Homology identity of proteins

* Single entry in transcriptome

**Supplementary Table 4.** Abbreviations in Figure 1, 3, 4 and 5

| **Abbreviations** | **Name** | **Abbreviations** | **Name** |
| --- | --- | --- | --- |
| G6P | Glucose-6-phosphate | PGmu | Phosphoglycerate mutase |
| F6P | Fructose-6-phosphate | PK | Pyruvate kinase |
| FBP | Fructose-1,6-bisphosphate | ALD | Aldolase |
| DHAP | Dihydroxyacetonephosphate | ENO | Enolase |
| GAP | Glyceraldehyde-3-phosphate | PDC | Pyruvate decarboxylase |
| BPG | 1,3-bisphosphoglycerate | RUBISCO | Ribulose-1,5-bisphosphate carboxylase/  oxygenase |
| 3PG | 3-phosphoglycerate | RUBP | Ribulose 1,5-bisphosphate |
| 2PG | 2-phosphoglycerate | CDPS | *ent*-copalyl diphosphate synthase |
| PEP | Phosphoenolpyruvate | *ent*-CPP | *ent*-copalyl pyrophosphate |
| PGP | Phosphoglycolate phosphatase | KS | Kaurene synthase |
| PGDH | 3-phosphoglycerate dehydrogenase | KO | Kaurene oxidase |
| GO | Glycolate oxidase | KH | Kaurene hydroxylase |
| GAT | Glycine amino transferase | MVA | Mevalonate |
| GD | Glycine decarboxylase | DXPS | 1-deoxy-D-xylulose-5-phosphate synthase |
| 3-PHP | 3-phosphohydroxypyruvate | DXPR | 1-deoxy-D-xylulose-5-phosphate reductoisomerase |
| DOXP | 1-deoxy-D-xylulose-5-phosphate | ISPD | 4-diphosphocytidyl-2C-methyl-D-erythritol synthase |
| MEP | 2-C-methyl-D-erythritol | ISPE | 4-diphosphocytidyl-2C-methyl-D-erythritol kinase |
| CDP-ME | 4-(CDP)-2-methyl-D-erythritol |  |  |
| CDP-MEP | 4-(CDP)-2-methyl-D-erythritol 2-phosphate | MECPS | 2-C-methyl-D-erythritol 2,4-cyclopyrophosphate synthase |
| CME-PP | 2-C-methyl-D-erythritol 2,4-cyclopyrophosphate | HDS | 1-hydroxy-2-methyl 2-(E)-butenyl-4-pyrophosphate synthase |
| HMBPP | 1-hydroxy-2-methyl 2-(E)-butenyl-4-pyrophosphate | ISPH | 1-hydroxy-2-methyl 2-(E)-butenyl-4-pyrophosphate reductase |
| IPP | Isopentenyl pyrophosphate | IPPI | Isopentenyl pyrophosphate delta-isomerase |
| DMAPP | Dimethylallyl pyrophosphate | AACT | Acetoacetyl-CoA thiolase |
| PSAT | 3-phosphoserine aminotransferase | HMGS | Hydroxymethylglutaryl-CoA synthase |
| PSP | 3-phosphoserine phosphatase | HMGR | Hydroxymethylglutaryl-CoA reductase |
| 3-PS | 3-phosphoserine | MVK | Mevalonate kinase |
| SHMT | Serine hydroxymethyltransferase | PMK | Phosphomevalonate kinase |
| SDC | Serine decarboxylase | PMD | Diphosphomevalonate decarboxylase |
| HMG CoA | 3-hydroxy-3-methylglutaryl coenzyme A | GGPPS | Geranyl geranyl pyrophosphate synthase |
| GGPP | Geranyl geranyl pyrophosphate | G6PI | Glucose-6-phosphate isomerise |
| PGAP | 3-phosphoglycerate phosphatase | PFK | Phosphofructokinase |
| GAD | glycerate dehydrogenase | TPI | Triosephosphate isomerase |
| AH-AT | alanine-hydroxypyruvate aminotransferase | GAPDH | Glyceraldehyde-3-phosphate dehydrogenase |
| 3PGA | 3-phosphoglycerate | PGK | Phosphoglycerate kinase |
| HK | Hexokinase |  |  |
|  |  |  |  |
